# Takotsubo syndrome with several hypertensive crises: an unexpected diagnosis

**DOI:** 10.1097/XCE.0000000000000291

**Published:** 2023-09-01

**Authors:** Małgorzata Niemiec, Nicola Dyrek, Klaudia Żądecka, Bartosz Gruchlik, Adrianna Berger-Kucza, Katarzyna Mizia-Stec

**Affiliations:** 1 First Department of Cardiology, Medical University of Silesia, Katowice, Poland

**Keywords:** cardiac involvement, hypertension, pheochromocytoma

## Abstract

We present an unusual clinical case of a 39-year-old woman admitted to the Department of Cardiology due to stenocardial pain accompanied by hypertensive crisis. The patient presented with severe chest pain and high blood pressure, along with a history of type 2 diabetes, hyperlipidemia, smoking, and hypertension. Initial tests showed elevated troponin T, glucose, CRP, and D-dimer levels, and electrocardiography and transthoracic echocardiography showed abnormalities suggesting acute myocardial infarction, but angiography did not reveal any significant coronary artery blockages. Further tests and imaging led to a diagnosis of takotsubo syndrome (TTS) and suspicion of pheochromocytoma, which was confirmed later biopsy. The presented case is very rare because the coexistence of TTS and pheochromocytoma is not common due to the rarity of the tumor. It is very important to make a quick and accurate diagnosis, because improperly treated cases can lead to death.

## Introduction

We would like to share an intriguing and unusual clinical case of a 39-year-old woman who presented to our Department of Cardiology with stenocardial pain and accompanying hypertensive crisis. The patient’s symptoms were suggestive of acute myocardial infarction (AMI) and therefore, the initial diagnosis was made accordingly. However, as the case progressed, the final diagnosis turned out to be quite unexpected.

## Case report

On admission, the patient complained of severe chest pain accompanied by symptoms associated with an increase in blood pressure (BP) to 210/110 mmHg. There was a stressful situation before admission to the hospital. In the history: type 2 diabetes, hyperlipidemia, nicotinism, arterial hypertension with accompanying spikes for 6 months and weight loss – 20 kg within 6 months. The patient was permanently on temisartan 40 mg and bisoprolol 2.5 mg.

**Fig. 1:**
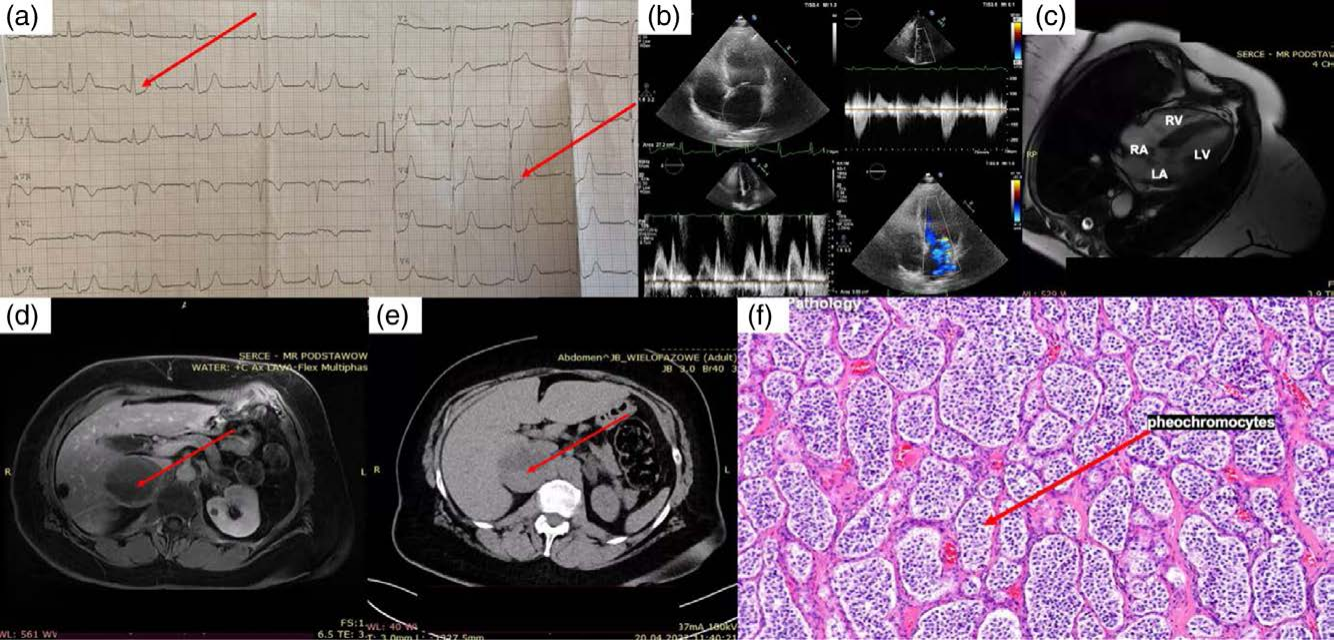
(a) ECG: depression of ST segments; (b) TTE on admission: mild mitral regurgitation, EF 37%, IVS 14–15 mm, LA 44 mm, LAA 27 cm^2^, EDD LV 50 mm, ESD LV 30 MM, edv 100 mL, ESV 63 mm, LVOT Vmax 2 m/s, VCI collapsed; (c) MRI of the heart: left ventricular hypertrophy to 17 with low LVOTO, LGE; (d) MR: a circular structure with heterogeneous signal is visible, probably coming from the right adrenal gland, 69 × 55 × 34; (e) CT of the abdominal cavity: a round, nodular lesion with a diameter of about 6 cm, with fairly smooth, regular contours, with a solid structure in its peripheral parts, in the inner parts of the lesion with a tissue density of 17–28 UH – thick fluid, after intravenous administration of the contrast, a slight enhancement is observed within the marginal parts of the tumor only in the venous phase of the examination, the tumor originates from the right adrenal gland; (f) Tumor biopsy: pheochromocytes, potassium dichromate stain.

Physical examination revealed a systolic murmur over the 3/6 apex and tenderness in the right hypochondrium. Laboratory tests showed an increase in the troponin T (0.6–1.1 ng/ml), glucose (300 mg%), CRP (27.5 mg/L) levels and D-dimers (910 ng/ml). Electrocardiography (ECG) showed ST segment depression in leads II and V4 (Fig. 1a). Transthoracic echocardiography showed a decreased left ventricular ejection fraction (37%) with segmental abnormalities of contractility of the basal segment of the inferior wall, IVS, posterior wall, and mild mitral regurgitation (Fig. 1b). A preliminary diagnosis was made – AMI, however, coronary angiography did not reveal any significant lesions in epicardial coronary arteries. Based on the above-mentioned results, diagnosis of takotsubo syndrome (TTS) was stated. During the hospitalization, the following were observed: numerous attacks of sudden dyspnea, BP spikes of 220/110, sinus tachycardia of 130/min, hand tremor, heavy sweating and anxiety. These seizures lasted a few minutes, and then the patient became weak. Holter-ECG showed: 17× atrial tachycardia 3–9×QRS up to 160/min. Cardiac MRI showed a dominant left ventricular hypertrophy up to 17 mm with a small left ventricular outflow tract and late gadolinium enhancement (Fig. 1c). Simultaneously, magnetic resonance showed a round structure with an inhomogeneous signal probably leading to the right adrenal gland (Fig. 1d). CT of the abdominal cavity were performed urgently, which showed: a focal lesion 6.2 cm in diameter with features of disintegration in the right lobe of the liver and a nodular lesion 6 cm in diameter with quite smooth and regular contours, which originated from the right adrenal gland. After intravenous contrast administration, a slight enhancement was observed in the marginal parts of the tumor in the venous phase (Fig. 1e). Pheochromocytoma was suspected. Measurements of methoxycatecholamines in urine were ordered: 3-methoxytyramine 2238 µg/day, metaphenrin and normetanephrine >limit of quantification. Due to the suspicion of pheochromocytoma, a biopsy could not be performed [1]. Doxazosin 2 × 2mg was added to the treatment. After a month, the tumor was operated on, and histopathological examinations confirmed the diagnosis of pheochromocytoma (Fig. 1f).

## Discussion

The case shows unusual presentation of phaeochromocytoma with baseline diagnostic process including AMI and TTS. Association between pheochromocytoma and TTS seems to be obvious, however, coexistence of these two diseases is rare because of rare frequency of the tumor [2,3]. It is very important to make the diagnosis as quickly and effectively as possible, because inadequately treated cases are fatal [4,5].

## Acknowledgements

### Conflicts of interest

There are no conflicts of interest.

## References

[1] Vanderveen KA Thompson SM Callstrom MR Young WF Jr Grant CS Farley DR. Biopsy of pheochromocytomas and paragangliomas: potential for disaster. Surgery 2009; 146:1158–1166. 19958944 10.1016/j.surg.2009.09.013

[2] Yalta K Yalta T Yetkin E. Pheochromocytoma and takotsubo syndrome: an ominous duo. Anatol J Cardiol 2022; 26:668–669. 35924295 10.5152/AnatolJCardiol.2022.2038 PMC9403880

[3] Yalta K Ozkan U Yalta T Yetkin E. Takotsubo syndrome in association with pheochromocytoma: clinical and practical considerations. Monaldi Arch Chest Dis 2021; 91. doi: 10.4081/monaldi.2021.1848. 10.4081/monaldi.2021.1848 34092074

[4] Manger WM Gifford RW. Pheochromocytoma. J Clin Hypertens (Greenwich) 2002; 4:62–72. 11821644 10.1111/j.1524-6175.2002.01452.x PMC8099329

[5] Farrugia FA Charalampopoulos A. Pheochromocytoma. Endocr Regul 2019; 53:191–212. 31517632 10.2478/enr-2019-0020

